# Acupuncture alleviates secondary brain injury in intracerebral hemorrhage-induced rats through activating the LKLF/Mfn2 pathway

**DOI:** 10.1590/1414-431X2026e15314

**Published:** 2026-08-17

**Authors:** Ying Kong, Xin Qu, Zhiwei Zhou, Qiyuan Tian, Hong Sun, Dawei Zhang, Yeqing Zhang, Zengxin Han

**Affiliations:** 1 Department of Acupuncture, The Second Affiliated Hospital of Heilongjiang University of Chinese Medicine, Harbin, China; 2 Department of Acupuncture, The First Affiliated Hospital of Jiamusi University, Jiamusi, China; 3 Department of Rehabilitation, Harbin Traditional Chinese Medicine Hospital, Harbin, China; 4 Department of Cardiology, First Affiliated Hospital, Heilongjiang University of Chinese Medicine, Harbin, China; 5 Department of Critical Care Medicine, The Second Affiliated Hospital of Heilongjiang University of Chinese Medicine, Harbin, China; 6 The Second Clinical Medical College, Heilongjiang University of Chinese Medicine, Harbin, China

**Keywords:** Acupuncture, Intracerebral hemorrhage, Mitochondrial, Oxidative stress, Apoptosis

## Abstract

Intracerebral hemorrhage (ICH) is a hemorrhagic stroke causing severe secondary brain injury. Acupuncture has been shown to be effective in treating ICH, but the mechanism remains unclear. ICH was induced by injection of autologous blood, followed by acupuncture treatment at *Baihui* (DU20) and *Qubin* (GB7) acupoints. Histopathological results showed that acupuncture inhibited ICH-induced neuronal apoptosis and oxidative stress in perihematomal areas. Importantly, molecular and pathological findings proved that acupuncture stimulation significantly induced the Lung-Kruppel-like factor (LKLF) expression in perihematomal tissues of ICH-induced rats. The embryonic day 18 (E18) rat primary cortical neurons were treated with hemin as an *in vitro* model, and LKLF was overexpressed using lentiviral vectors to ascertain its function. LKLF overexpression inhibited neuronal apoptosis and oxidative stress and mitigated mitochondrial injury evidenced by decreased mitochondrial membrane potential in hemin-induced neurons. Both *in vivo* and *in vitro* experiments showed that mitofusin2 (Mfn2) expression was inhibited in neurons but increased upon acupuncture and LKLF overexpression. This suggested that the remission of ICH injury via acupuncture or LKLF overexpression might be related to the upregulation of Mfn2 reactivity. Dual luciferase assay demonstrated that LKLF activated Mfn2 promoter activity, showing that LKLF might improve mitochondrial function through transcriptional activation of Mfn2 expression, which subsequently mitigated hemin-induced neuronal injury. The rescue experiments *in vivo* indicated that LKLF knockdown suppressed Mfn2 expression and counteracted the attenuation of acupuncture on ICH-induced neuronal injury. These findings suggested that acupuncture might activate the LKLF/Mfn2 pathway, therefore inhibiting oxidative stress and neuronal apoptosis in ICH-induced rats.

## Introduction

Intracerebral hemorrhage (ICH) refers to bleeding caused by the rupture of a non-traumatic blood vessel within the brain. It results in blood infiltrating the brain tissue, leading to brain damage and eventual disability or death (1). Perihematomal edema due to blood-brain barrier dysfunction after ICH is one of the most common and lethal consequence of this condition (2). Perihematomal edema aggravates ICH-induced secondary brain injury (SBI), which ultimately results in neurological deficits and neuronal death (3). The mechanism of SBI is intricate, mostly encompassing oxidative stress, mitochondrial dysfunction, and neuronal death (including apoptosis and necrosis) (4). Currently, effective therapeutic alternatives for ICH are scarce, and few pharmacologic or surgical interventions provide significant benefit. Conventional treatment and comprehensive management could reduce ICH mortality, but they also result in severe neurological impairment. Acupuncture has been extensively employed in the treatment of ICH and other neurological disorders, effectively enhancing patient outcomes (5). However, the mechanism by which acupuncture operates in the treatment of ICH remains inadequately elucidated.

Krupple-like factors (KLFs) are a family of zinc finger transcription factors that are crucial in the regulation of cell apoptosis, metabolism, and other biological processes (6). The Krupple family of transcription factors consists of 17 highly conserved members (KLF1-17), of which Lung-Kruppel-like factor (LKLF) has been shown to play an important role as a protective factor in diseases such as cerebrovascular dysfunction (7). Significantly, Lu et al. (8) revealed that LKLF overexpression ameliorated the SBI induced by ICH in rats. Nevertheless, the mechanism remains unclear.

Mitochondria are highly dynamic organelles, and the dynamic regulation of mitochondrial membrane fusion and fission is crucial for sustaining normal mitochondrial function (9). Numerous pathological disorders, including neurodegenerative diseases, have been linked to the dysregulation of mitochondrial dynamics (10). Mitofusin2 (Mfn2) is a GTPase embedded in the outer mitochondrial membrane and has a role in the preservation and operation of the mitochondrial network (11). Mfn2 overexpression alleviated mitochondrial dysfunction by promoting mitochondrial fusion, whereas Mfn2 knockdown impaired mitochondrial function and aggravated apoptosis by inducing mitochondrial fission (12). More importantly, the elevation of Mfn2 expression attenuated oxidative stress and neuronal apoptosis in subarachnoid hemorrhage rats (13). Consequently, the regulation of Mfn2 expression may also be involved in the regulation of mitochondrial dysfunction after ICH, thereby alleviating the SBI. Studies have shown that KLFs are involved in Mfn2-mediated regulation of mitochondrial function (14). Therefore, we hypothesized that LKLF may be involved in the regulation of mitochondrial function by modulating Mfn2 expression, thereby attenuating the SBI caused by ICH. Previous studies have shown that acupuncture treatment promoted the expression of LKLF (15), but this phenomenon has not been reported in ICH. Consequently, we considered whether acupuncture alleviated mitochondrial injury by modulating LKLF expression, thereby mitigating the neuronal injury induced by ICH.

In this study, an *in vivo* rat ICH model and an *in vitro* embryonic day 18 (E18) rat primary cortical neuron model were used to investigate the role of the LKLF/Mfn2 pathway in ICH. We speculated that acupuncture inhibits SBI in ICH rats and hemin-induced neuronal injury by activating the LKLF/Mfn2 pathway. Our findings might elucidate the molecular mechanism by which acupuncture ameliorated the SBI in ICH rats.

## Material and Methods

### Animals

Male Sprague-Dawley (SD) rats aged 8 weeks were housed in a controlled environment with a temperature of 22±1°C and humidity of 45-55%. Appropriate (12-h light/dark) circadian light cycles were provided. The rats were free to access adequate food and water for 1 week before the experiment. A total of 72 rats were used for statistics in this study. All rats were obtained from Changsheng Biotechnology Co. (China).

The manuscript does not contain clinical studies or patient data. The animal protocols were approved by the Animal Experimental Ethical Inspection of Heilongjiang University of Chinese Medicine (Affidavit of Approval of Animal Use Protocol No. 2024062814).

### Induction and treatment of ICH rats

As previously described (16), an ICH rat model was established by injecting 50 μL autologous blood into the right striatum of each rat. Rats were anesthetized by inhalational 3% isoflurane (maintenance: 2% isoflurane), mounted onto a stereotactic apparatus, and the scalp was exposed. A small hole was then drilled in the right striatum of rats (0.2 mm right to the anterior fontanel and 3.5 mm lateral to the midline). The autologous blood, which was collected from the tail vein, was injected slowly (6 mm vertical to the cortical surface, 25 μL/min) into the hole using a microliter syringe. The needle was maintained in place for 5 min to prevent reflux. Finally, the scalp was sealed. The sham operation was conducted without injection.

**Figure 1:**
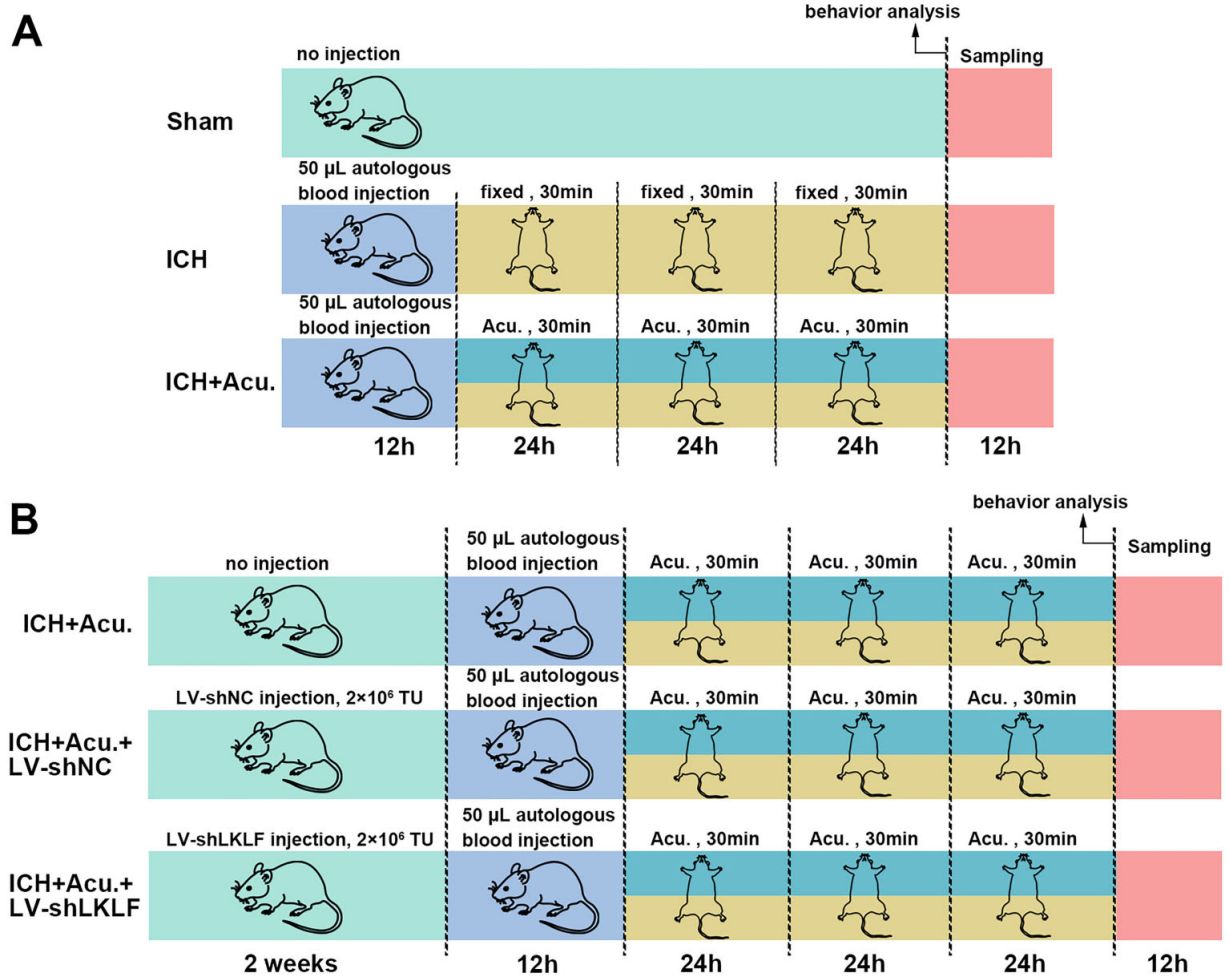
Flowchart of animal modeling. **A**, The intracerebral hemorrhage (ICH) model was established by injecting 50 μL of autologous blood into the right striatum of rats. Acupuncture (Acu.) treatment was performed every 24 h for 30 min, three times in total. Rats in the ICH group were only restrained and fixed for 30 min without acupuncture treatment. Rats in the sham group were only subjected to needle insertion without autologous blood injection. **B**, Two weeks before ICH modeling, lentivirus-mediated shRNA targeting LKLF (LV-shLKLF) or negative control lentiviral shRNA vector (LV-shNC) was injected. The procedures for ICH modeling and acupuncture were the same as described above.

*Baihui* (DU20)-penetrating *Qubin* (GB7) acupuncture has been extensively demonstrated to be effective in the treatment of ICH (17,18). Rats were treated with acupuncture 12 h postoperatively according to a previous study (16). Acupuncture was initiated at the *Baihui* acupoint (DU20, located midway between both ears), with the needle inserted subcutaneously toward the *Qubin* acupoint (GB7, at the leading edge of the tragus). The needle was then manually rotated at a speed of 200 turns per minute, alternating between clockwise and counterclockwise directions (19). Acupuncture treatment was performed every 24 h for 30 min, three times in total. Rats were sacrificed by CO_2_ inhalation 12 h after the last acupuncture treatment, and perihematomal tissues were collected (Figure 1A). Tissues from one set of rats were used for molecular biology analyses, including real-time PCR, Western blot, and commercial kit assays. Perihematomal tissues collected from another set of rats were used for all histopathological analyses.

To assess the LKLF expression in rats, 5 μL lentivirus (LV-shLKLF or LV-shNC, 4.0×10^8^ TU/mL, 0.2 μL/min) was injected into the right striatum of each rat 2 weeks before ICH induction (Figure 1B).

### Cell culture and treatment

The E18 rat primary cortical neuron culture has been described in detail in previous literature (20), and the cells were free of mycoplasma contamination. Briefly, cortical tissue from fetal rat brains was removed under a dissecting microscope (Olympus, Japan) and digested using 0.25% pancreatin (Sigma, USA) at 37°C for 20 min, with gentle agitation every 10 min. Dissociated cells were resuspended as a single-cell suspension in Neurobasal medium (Gibco, USA) supplemented with 2% B27 (Gibco) and 0.5 mM GlutaMAX (Gibco). The cells were then plated onto a 24-well plate precoated with poly-D-lysine (100 µg/mL) (MedMol, China) and maintained at 37°C in 5% CO_2_. The medium was replaced after 8 h. After that, half of the medium was replaced with fresh medium every 36 h. Cells were harvested at day *in vitro* 7 for subsequent experiments.

To overexpress LKLF in primary cortical neurons, cells were infected with lentiviral vector (LV-oeLKLF) and control vector (LV-vector). After 48 h, the cells were treated with hemin (100 μM) (Macklin, China) for 24 h. The cells were then collected for follow-up detection.

### Immunohistochemical (IHC) and Nissl staining

Brain tissue was fixed by immersion in 4% paraformaldehyde, followed by washing in PBS before processing for paraffin embedding. Perihematoma tissues under different conditions were embedded in paraffin and subsequently sliced (5-μm thick). For IHC staining preparation, slices were incubated with primary antibodies targeting LKLF (1:100, #DF13602, Affinity, China) at 4°C overnight. Slices were then washed three times with PBS and incubated with the secondary antibody (Goat anti-Rabbit IgG, 1:500, #31460, ThermoFisher, USA). The staining result was visualized using DAB solution (MXB Biotechnologies, China). After counterstaining with hematoxylin (Solarbio, China), slices were examined using a microscope (Olympus, Japan). For Nissl staining, brain slices were stained with 0.5% cresyl violet (Sinopharm, China) at room temperature for 10 min and observed at 400× magnification.

### Dihydroethidium (DHE) and TUNEL staining

To detect the ROS levels in tissues around the hematoma in rats, slices (10-μm thick) were stained with DHE solution (1:100) for 30 min at 37°C in the dark according to the DHE staining kit (Beyotime, China). Following the ROS assay (KeyGEN, China), cells with different treatments were incubated with DHE (10 μM) for 30 min at 37°C. Fluorescent images were obtained using a fluorescence microscope (Olympus, Japan) at 200× magnification.

TUNEL staining was performed to assess cell apoptosis *in vivo*. Cells were permeabilized with 0.1% Triton X-100 (Beyotime) and then cultured in a dark and humid environment at 37°C for 1 h using TUNEL reaction solution (Roche, Switzerland).

### Tetrachloro-tetraethylbenzimidazol carbocyanine iodide (JC-1) staining

The mitochondrial membrane potential of neurons was detected by JC-1 staining. According to the assay kit protocol (Biosharp, China), pretreated neurons were incubated with JC-1 working solution (1 mL) per sample at 37°C for 20 min. Neurons were then washed twice using JC-1 staining buffer. Finally, neurons were observed under a fluorescent microscope at 400× magnification.

### Malondialdehyde (MDA) and glutathione (GSH) measurement

MDA is the final stable product of lipid peroxidation and GSH is an important endogenous antioxidant. MDA and GSH were detected to indicate the degree of oxidative stress injury (21).

Brain tissues around the hematoma from different groups were collected, lysed, and centrifuged (1:9, w/v; 663 *g*; 10 min; 4°C). The supernatant was collected for determination of MDA and GSH contents in brain tissues. Cell precipitates were resuspended with PBS, lysed by sonication, and the cell suspensions were collected for detection of MDA and GSH contents. Following the protocols provided by the ELISA kits (Jiancheng Bioengineering Institute, China), the levels of MDA and GSH were evaluated.

### Dual-luciferase reporter assay

The pheochromocytoma (PC12) cells (Research Resource Identifier (RRID): CVCL_0481) were cultured in a complete DMEM medium (Servicebio, China) supplemented with 5% FBS (Tianhang, China) and 10% equine serum (Solarbio) in a 5% CO_2_ incubator at 37°C. The experiments were performed with mycoplasma-free PC-12 cells, as confirmed using a mycoplasma detection PCR assay. We detected luciferase activity through Dual-Luciferase¯ Reporter Assay System (KeyGEN) and calculated the ratio of Renilla luciferase to firefly luciferase activity. PC12 cells were co-transfected with luciferase reporter plasmid (pGL3-Mfn2 promoter, pRL-TK) and LKLF overexpression plasmid. pGL3-vector was used as the empty control. These vectors were transfected using Lipofectamine 3000 (Invitrogen, USA) after cell adhesion. Cells were lysed 48 h after transfection, and then the firefly and Renilla luciferase reaction solutions were prepared and incubated with the cell lysate at room temperature. A total of 20 μL cell lysate was added to 100 μL firefly luciferase reaction solution and mixed with a pipette, and the firefly luciferase activity was determined. Then, a total of 100 μL of Renilla luciferase reaction solution was added, and the activity of Renilla luciferase was detected. Renilla luciferase activity was normalized to that of firefly luciferase.

### Western blotting

Both the rat tissues around the hematoma and neurons with different treatments were lysed on ice using RIPA lysis buffer (Solarbio). The supernatant from each sample was collected after centrifugation at 10,000 *g* at 4°C for 5 min. Subsequently, protein concentrations were measured using a BCA protein assay kit (Solarbio). Equal protein samples were separated in SDS-PAGE and transferred to the PVDF membrane (Millipore, USA), which was then blocked with the blocking buffer (Solarbio). Membranes were incubated with primary antibodies overnight at 4°C and then with HRP-conjugated secondary antibodies for 1 h at room temperature. Finally, protein bands were revealed using an ECL kit (Solarbio). The titers of antibodies were as follows: LKLF antibody (1:500, #DF13602, Affinity), Mfn2 antibody (1:1000, #12186-1-AP, Proteintech, China), GAPDH antibody (1:10000, #60004-1-Ig, Proteintech), Goat anti-Rabbit IgG antibody (1:3000, #SE134, Solarbio), and Goat anti-Mouse IgG antibody (1:3000, #SE131, Solarbio).

### Real-time fluorescence quantitative polymerase chain reaction

Total RNA from rat tissues around the hematoma and neurons under different conditions was extracted using a TRIpure reagent (BioTeke, China). Then, the single-stranded cDNA was synthesized from RNA using All-in-One First-Strand SuperMix (Magen, China). Real-time PCR was performed using the SYBR Green mix (Solarbio) on a qPCR system. mRNA expression was determined using the 2^-ΔΔCt^ method. The forward primer of LKLF was 5′-CTCAGCGAGCCTATCTTGCC-3′. The reverse primer of LKLF was 5′-CCAGTCCCATGGACAGGATG-3′. The forward primer of Mfn2 was 5′-CTCTATGGGCATTCTCGT-3′. The reverse primer of Mfn2 was 5′-CTGGCATACTCCACAAAC-3′.

### Statistical analysis

All data are reported as means±SD. Groups were compared with one-way analysis of variance (ANOVA) followed by Tukey's test using GraphPad Prism 9 software (USA). Statistical significance was set at P<0.05.

## Results

### Acupuncture alleviated neuronal injury and oxidative stress and promoted LKLF expression in rats after ICH

**Figure 2:**
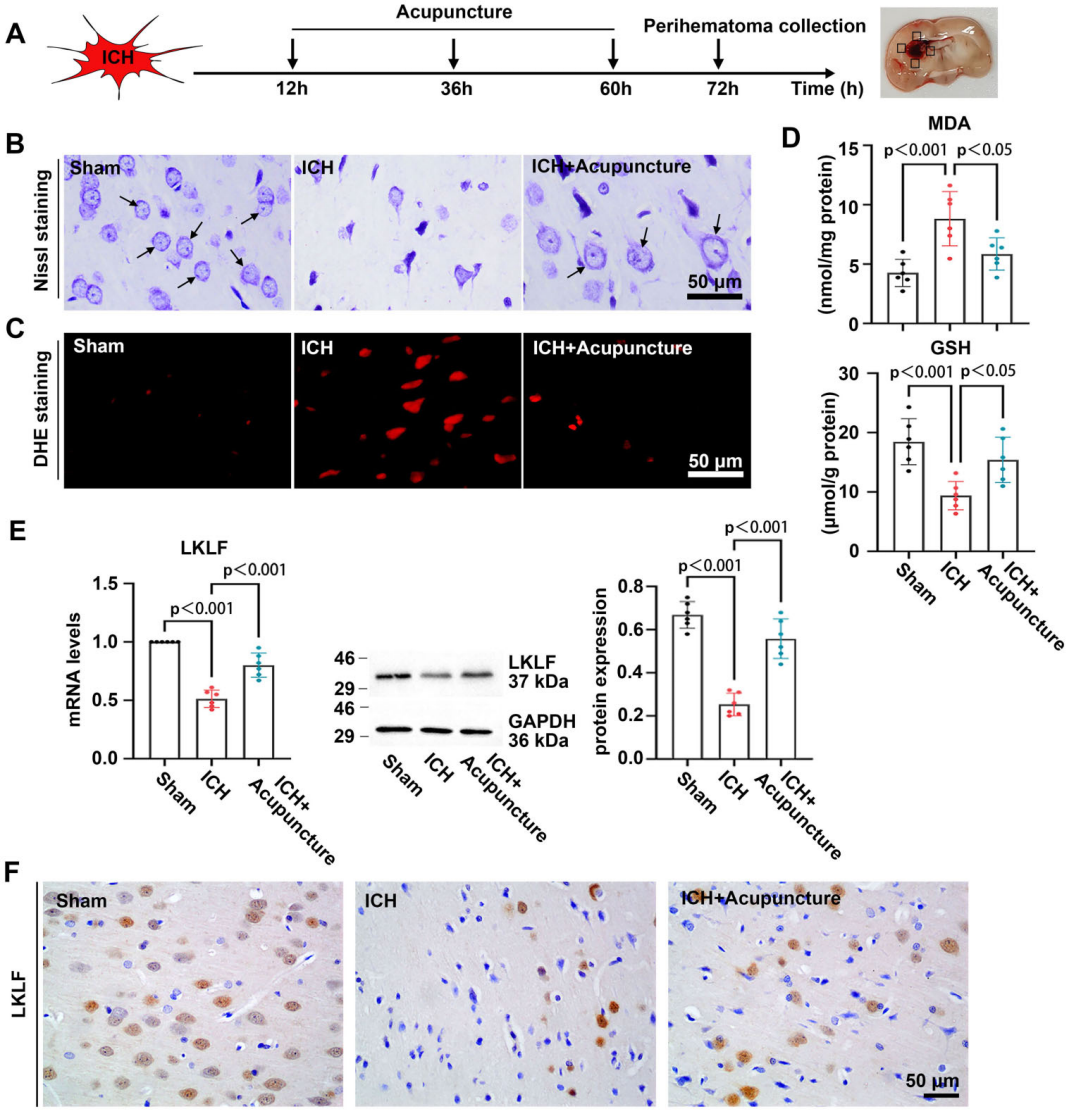
Acupuncture alleviated neuronal apoptosis and oxidative stress and promoted Lung-Kruppel-like factor (LKLF) expression in intracerebral hemorrhage (ICH) rats. **A**, Simple flow chart of ICH rat model establishment and experimental design. The right side was the location for tissue sampling around the hematoma. **B**, Representative photomicrographs of perihematoma in rats from different groups using Nissl staining (scale bar: 50 μm). Black arrows indicated Nissl-positive neurons. **C**, Representative fluorescence images of DHE staining in perihematoma of rats in different groups (scale bar: 50 μm). **D**, Quantification of malondialdehyde (MDA) and glutathione (GSH) levels in perihematoma of rats from different groups. **E**, The mRNA levels of LKLF and representative western blotting bands and quantification of protein levels of LKLF around the hematoma in rats from different groups. GAPDH served as a loading control. **F**, Representative immunohistochemical images of LKLF around hematoma in rats from different groups (scale bar: 50 μm). Data are reported as mean and SD; ANOVA.

To elucidate the effect of acupuncture on brain injury after ICH, we constructed an ICH-induced rat model and acupuncture was administrated 12 h post-surgery (Figure 2A). The survival of neurons around the hematoma in ICH rats was visualized based on Nissl staining. Nissl-positive cells were significantly decreased in ICH rats compared with the sham group, while the number of Nissl bodies was increased in ICH rats with acupuncture treatment (Figure 2B). This indicated that acupuncture inhibited neuronal injury in ICH-induced rats. Acupuncture therapy also decreased the ROS and MDA levels and increased the GSH expression in perihematomal tissues of ICH rats (Figure 2C and D). These data showed that acupuncture suppressed oxidative stress activation in rats after ICH. Moreover, we found that LKLF expression was significantly decreased in ICH-induced rat tissues around the hematoma, which was elevated after acupuncture treatment (Figure 2E and F). Hence, these data suggested that acupuncture therapy alleviated neuronal injury and oxidative stress, and restored LKLF expression in ICH rats.

### LKLF overexpression inhibited neuronal apoptosis in E18 rat primary cortical neurons induced by hemin

**Figure 3:**
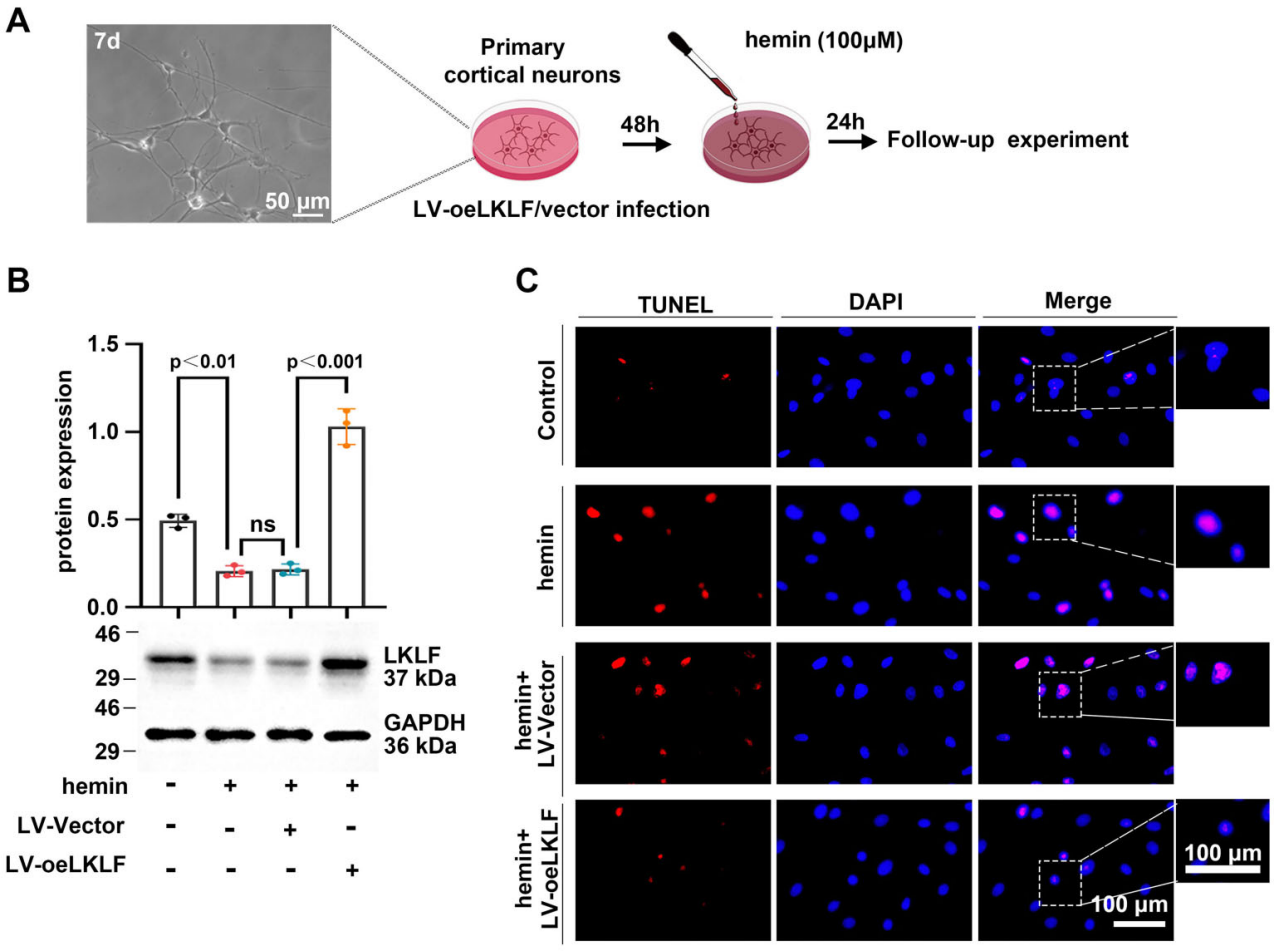
Lung-Kruppel-like factor (LKLF) overexpression inhibited neuronal apoptosis in primary cortical neurons induced by hemin. **A**, The embryonic d 18 (E18) rat primary cortical neurons were infected by lentiviral vectors (LV-Vector or LV-oeLKLF) for 48 h and then treated with hemin (100 μM) for 24 h. Subsequent experiments were then performed. On the left is a representative image of primary cortical neurons cultured for 7 days (scale bar: 50 μm). **B**, Representative western blot bands of LKLF and the quantitative results in primary cortical neurons with different treatments. GAPDH served as a loading control. **C**, Representative TUNEL staining images in primary cortical neurons from different groups (scale bar: 100 μm). LV: lentiviral vector; LV-Vector: empty control; LV-oeLKLF: LKLF overexpression. Data are reported as mean and SD; ANOVA. ns: not significant.

Appropriate hemin concentrations were used to induce neuronal injury in primary cortical neurons (22) to further investigate the role of LKLF. E18 rat primary cortical neurons with LKLF overexpression were stimulated by hemin for *in vitro* assays (Figure 3A). The protein levels of LKLF were obviously decreased in hemin-induced neurons, and LKLF overexpression via lentiviral infection successfully increased LKLF expression (Figure 3B). TUNEL-positive neurons in the hemin-induced group were significantly increased compared to the control group, which were remarkably decreased after LKLF overexpression (Figure 3C). These data indicated that LKLF overexpression significantly inhibited neuronal apoptosis in hemin-induced primary cortical neurons.

### LKLF overexpression inhibited oxidative stress and mitochondrial damage in E18 rat primary cortical neurons induced by hemin

**Figure 4:**
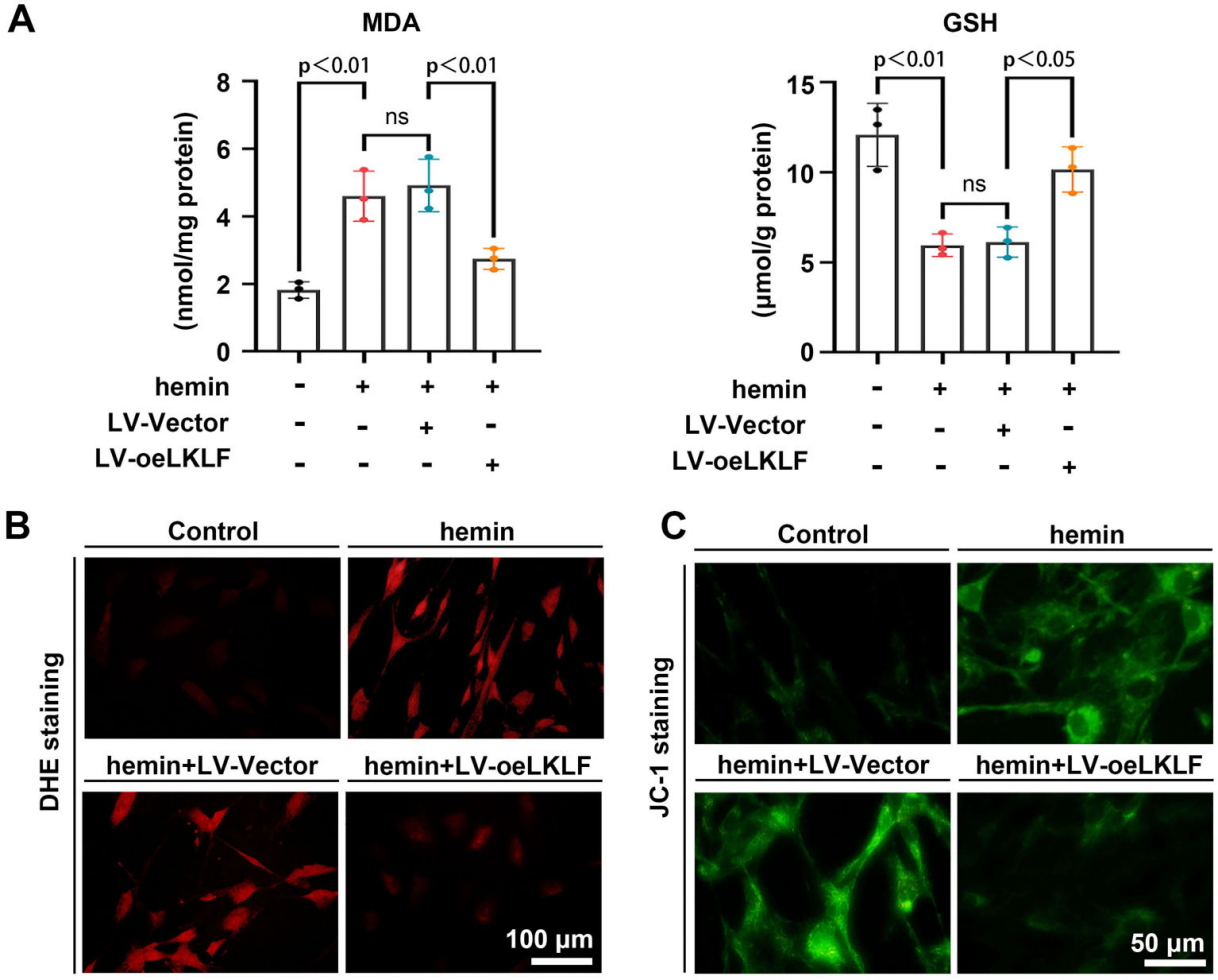
Lung-Kruppel-like factor (LKLF) overexpression inhibited oxidative stress in hemin-induced primary cortical neurons and promoted mitochondrial function. **A**, Levels of malondialdehyde (MDA) and glutathione (GSH) in primary cortical neurons under different conditions. **B**, Representative DHE staining images of primary cortical neurons under different conditions (scale bar: 100 μm). **C**, Representative tetrachloro-tetraethylbenzimidazol carbocyanine-iodide (JC-1) staining images of primary cortical neurons with different treatments (scale bar: 50 μm). LV: lentiviral vector; LV-Vector: empty control; LV-oeLKLF: LKLF overexpression. Data are reported as mean and SD; ANOVA. ns: not significant.

Next, we detected the effect of LKLF in oxidative stress. MDA and ROS levels were significantly increased in primary cortical neurons after hemin stimulation, which were decreased after LKLF overexpression. Overexpressing LKLF also reversed the GSH levels in hemin-induced neurons (Figure 4A and B). This suggested that LKLF overexpression inhibited oxidative stress in hemin-induced neurons. At the same time, we evaluated the effect of LKLF on mitochondrial damage in hemin-induced neurons. The opening of the mitochondrial membrane permeability transition pore (MPTP) results in mitochondrial injury (23). MPTP opening was assessed by fluorescence intensity of JC-1 staining. The fluorescence in hemin-induced neurons was enhanced, which was obviously decreased after LKLF overexpression (Figure 4C). This indicated that LKLF overexpression alleviated mitochondrial damage in hemin-induced neurons. Together, these data suggested that overexpressing LKLF inhibited oxidative stress and mitochondrial injury in hemin-stimulated primary cortical neurons.

### LKLF promoted the expression of Mfn2 in E18 rat primary cortical neurons

**Figure 5:**
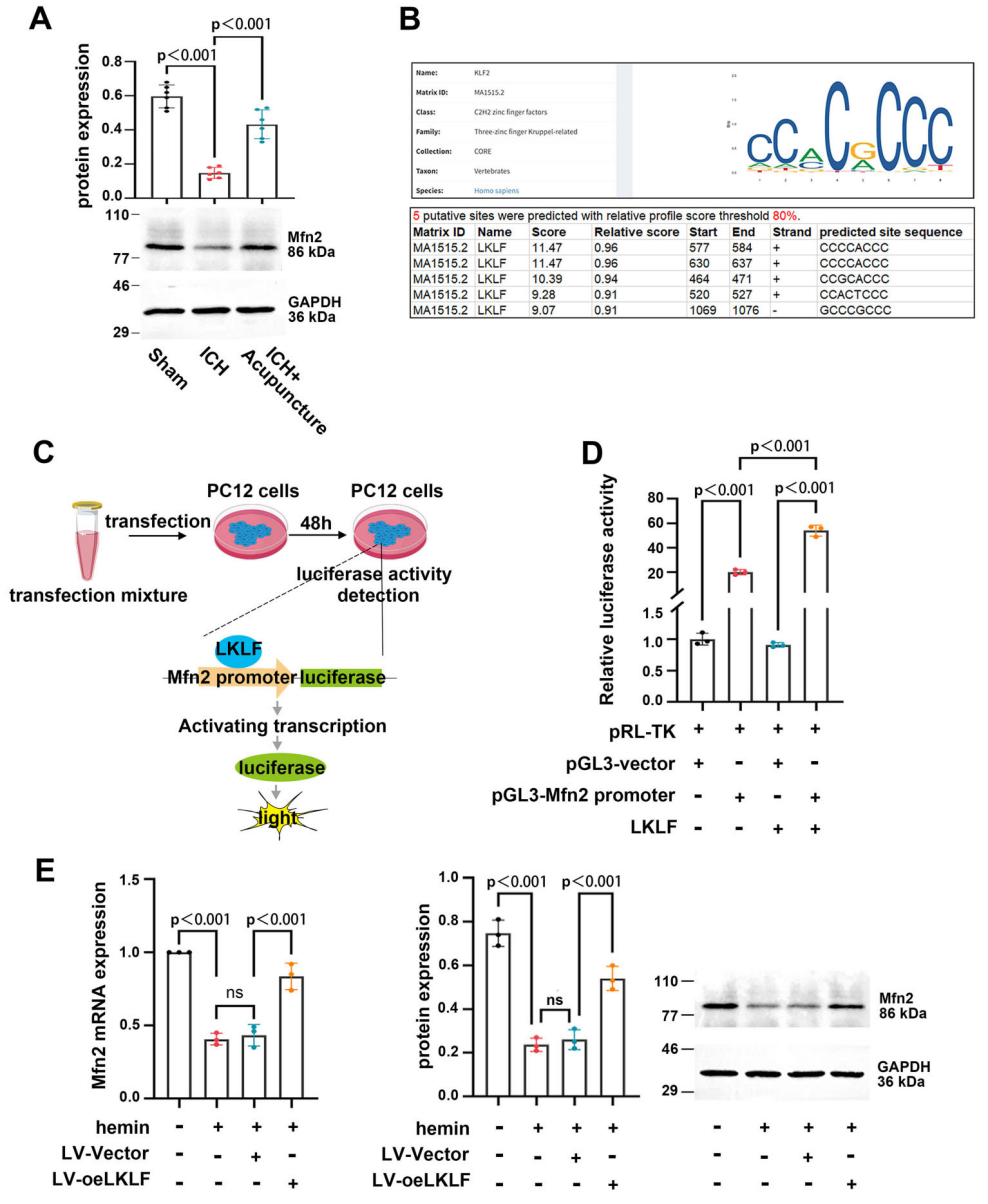
Lung-Kruppel-like factor (LKLF) promoted the expression of mitofusin2 (Mfn2) in primary cortical neurons of rats. **A**, Representative western blot bands and quantitative results of Mfn2 around the hematoma in rats from different groups. GAPDH served as a loading control. **B**, The JASPAR website (https://jaspar.elixir.no/) predicted the binding sites of the promoter sequences of LKLF and Mfn2. **C**, Luciferase reporter vector containing Mfn2 promoter sequence and LKLF overexpression plasmid were co-transfected into PC12 cells, and luciferase activity was detected after 48 h. **D**, Luciferase reporter assay demonstrated direct interaction of LKLF with the promoter of the Mfn2 gene. **E**, The mRNA and protein levels of Mfn2 and quantitative results of protein levels in primary cortical neurons with different treatments. LV: lentiviral vector; LV-Vector: empty control; LV-oeLKLF: LKLF overexpression; pRL-TK: Renilla luciferase reporter vector; pGL3: firefly luciferase reporter vector. Data are reported as mean and SD; ANOVA. ns: not significant.

Mfn2 plays an important role in maintaining mitochondrial function (12). A significant decrease of Mfn2 expression was observed in perihematomal tissues of ICH rats, and the levels of Mfn2 were elevated after acupuncture treatment (Figure 5A). We further explored whether LKLF overexpression alleviated mitochondrial damage by regulating Mfn2 expression. The Mfn2 promoter contains multiple LKLF binding sites, as indicated by the JASPAR website (Figure 5B). The LKLF overexpression plasmid and pGL3-luciferase reporter constructs containing Mfn2 promoter sequence were co-transfected into PC12 cells and then luciferase activity was detected 48 h after transfection (Figure 5C). LKLF significantly activated the promoter activity of Mfn2 (Figure 5D). Moreover, hemin-induced reduction of Mfn2 expression in neurons of rats was also reversed by LKLF overexpression (Figure 5E). These results revealed that LKLF promoted the expression of Mfn2 in primary cortical neurons.

### Acupuncture mitigated neural apoptosis and oxidative stress in ICH rats by activating the LKLF/Mfn2 pathway

**Figure 6:**
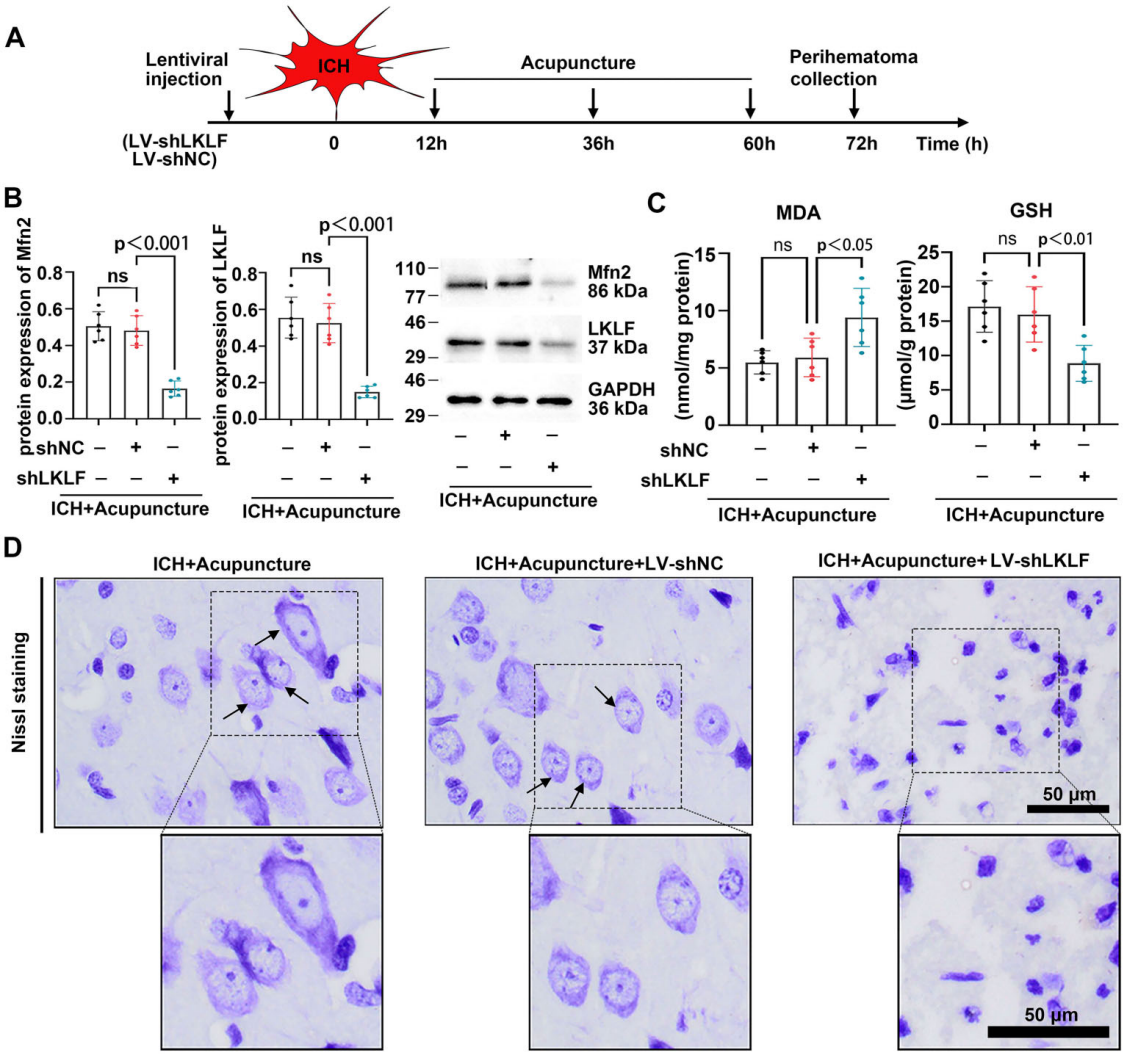
Acupuncture mitigated neural apoptosis and oxidative stress in intracerebral hemorrhage (ICH) rats by activating the LKLF/Mfn2 pathway. **A**, Simple flow chart of ICH rat model with LKLF knockdown establishment and experimental design. **B**, Protein levels and quantitative results of Mfn2 and LKLF in perihematoma of rats among different groups. **C**, Malondialdehyde (MDA) and glutathione (GSH) levels in perihematoma of rats under different conditions. **D**, Representative Nissl staining images around the hematoma in rats from different groups (scale bar: 50 μm). Black arrows indicate Nissl-positive neurons. LKLF: Lung-Kruppel-like factor; Mfn2: mitofusin2; LV: lentiviral vector; LV-shNC: empty control; LV-shLKLF: LKLF knockdown. Data are reported as mean and SD; ANOVA. ns: not significant.

Acupuncture and LKLF overexpression have been shown to alleviate neuronal apoptosis and oxidative stress *in vivo* and *in vitro*, while LKLF was proven to regulate Mfn2 expression in rat primary cortical neurons. Next, we further investigated whether acupuncture inhibited neuronal injury through the LKLF/Mfn2 pathway. We constructed an ICH rat model and knocked down LKLF two weeks before modeling. Rats received acupuncture treatment 12 h after ICH induction (Figure 6A). The expression of Mfn2 was also downregulated in ICH rats with LKLF knockdown (Figure 6B). LKLF knockdown caused a significant decrease in Nissl-positive neurons, indicating severe neural apoptosis in ICH rats (Figure 6C). Knocking down LKLF also exacerbated oxidative stress in rats after ICH, evidenced by increased MDA and decreased GSH (Figure 6D). These data showed that acupuncture inhibited neural injury in ICH rats through the LKLF/Mfn2 pathway activation.

## Discussion

The pathology of ICH is highly intricate. ICH-induced hematoma diminishes local tissue blood supply, causing secondary ischemia-reperfusion, which causes mitochondrial dysfunction and initiates a series of cellular signals that ultimately culminate in neuronal death. Acupuncture therapy has demonstrated a beneficial therapeutic impact on ICH in clinical cases or experimental animal models (24,25). In this study, ICH was induced in rats by autologous blood injection to investigate the protective effects and mechanism of acupuncture on neuronal apoptosis, oxidative stress, and mitochondrial dysfunction. ICH increased generation of ROS, aggravated oxidative stress, and caused neuronal apoptosis (21). Acupuncture has been demonstrated to inhibit neuronal apoptosis and oxidative stress during ICH (26,27), which is consistent with our findings. The normal expression of KLFs plays a crucial role in regulating neuronal development and regeneration. The dysregulation of KLFs is associated with a variety of neurological diseases (28). LKLF expression was found to be downregulated in ICH rats (8) and acupuncture treatment upregulated LKLF expression in rats with myocardial ischemia-reperfusion injury (15). Our results similarly demonstrated that LKLF was significantly down-regulated in the brain tissue of rats after ICH, which was upregulated after acupuncture treating. This suggested that the efficacy of acupuncture treatment on ICH-induced SBI might rely on the upregulation of LKLF expression.

Oxidative stress plays an important role in the progression of ICH-induced SBI. Following ICH induction, large amounts of ROS are released, resulting in massive membrane lipid peroxidation and oxidative damage to proteins and DNA. Studies have shown that the stimulation of LKLF expression reduces oxidative stress levels in coronary microvascular dysfunction (29). Up-regulation of LKLF inhibited ROS production in response to oxidative stress injury in atherosclerotic disease (30). In this study, we found that the overexpression of LKLF promoted hemin-induced antioxidant effects in neurons, marked by reduced ROS and MDA levels and increased GSH levels in neuronal cells. This suggested that LKLF overexpression effectively attenuated hemin-induced oxidative stress in neurons. The neuronal apoptosis next to the hematoma is a significant mechanism of neurological injury after ICH (31). Numerous factors induce neuronal apoptosis after ICH, such as free radical cascade reaction, inflammatory response, and cytokine stimulation (32). A recent study indicated that ROS mediated multiple forms of neuronal death during ICH, including apoptosis (33). Our study suggested that hemin triggered neuronal apoptosis, while LKLF overexpression inhibited this process, potentially due to its antioxidant effect. Thus, the upregulation of LKLF expression effectively inhibited neuronal oxidative damage induced by ICH or hemin.

Mitochondria serve as the locus for intracellular oxygen radical production and are the primary target of these radicals. Mitochondrial dysfunction directly impairs the activity of oxidative phosphorylation and the electron transport chain, leading to an increased generation of ROS. ROS overproduction induces oxidative stress, resulting in metabolic abnormalities that precipitate a cascade of pathogenic alterations, ultimately leading to cell apoptosis and oxidative damage. Our *in vitro* experiments were aligned with studies showing that mitochondrial dysfunction was induced in *in vitro* and *in vivo* ICH models (34,35). Research indicates that after ICH, the facilitation of mitochondrial fusion significantly reduces mitochondrial ROS production and neuronal apoptosis in experimental animal models, while also attenuating the severity of brain damage (36). The mitochondrial fusion protein Mfn2 facilitates mitochondrial fusion and preserves normal mitochondrial function (37). Mfn2 deficiency has been demonstrated to provoke ROS accumulation and exacerbate cellular or tissue damage across several disorders (38,39). Mfn2 overexpression alleviated oxidative damage and cell apoptosis in subarachnoid hemorrhage (13) and other diseases. Our findings demonstrated that Mfn2 expression was downregulated following neuronal damage both *in vivo* and *in vitro*. This may be responsible for the induction of mitochondrial dysfunction after neuronal injury. Acupuncture treatment upregulated Mfn2 expression in rats with ICH reperfusion injury (40), similar to our results. This indicated that the inhibition of ICH-induced injury by acupuncture may be related to the up-regulation of Mfn2 expression.

Zhang et al. (14) suggested that KLF family members regulate Mfn2 expression and improve mitochondrial function. This prompted us to investigate the relationship between LKLF and Mfn2. LKLF was shown to activate Mfn2 promoter activity in PC12 cells in the dual luciferase assays, and the Mfn2 expression was increased after LKLF overexpression in hemin-induced neurons. However, LKLF suppression counteracted the protective effect of acupuncture against neuronal oxidative damage and inhibited Mfn2 expression in ICH rats. This indicated that acupuncture might maintain normal mitochondrial function by activating the LKLF/Mfn2 pathway, thereby alleviating neuronal injury in rats after ICH.

**Figure 7:**
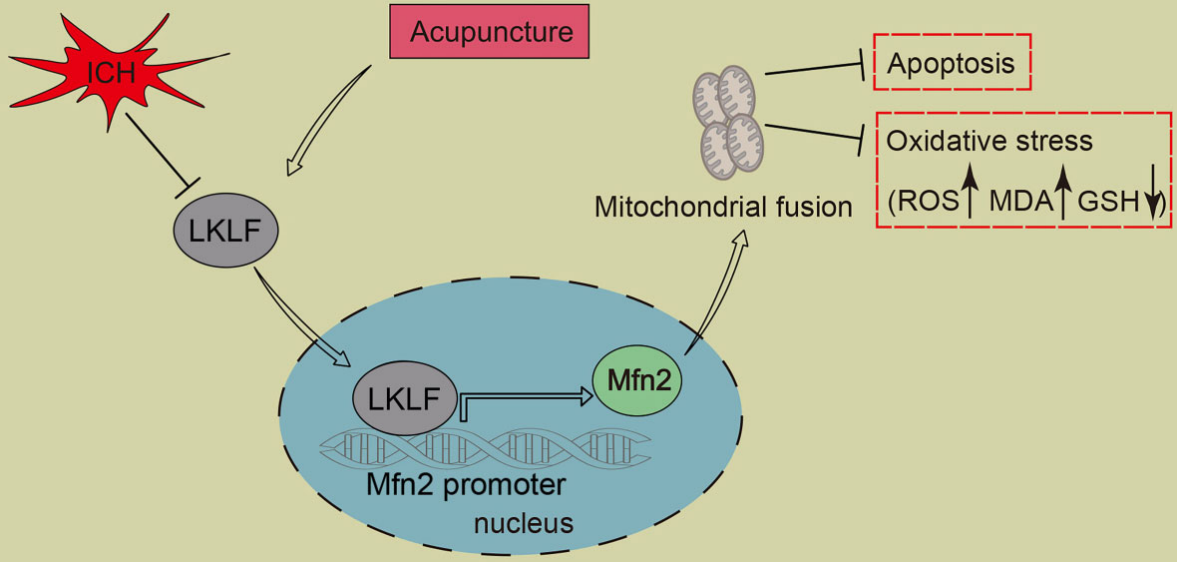
Acupuncture alleviated intracerebral hemorrhage (ICH) injury via LKLF/Mfn2 pathway. Acupuncture stimulated LKLF expression, which activated Mfn2 transcriptional activity and alleviated mitochondrial injury and then reduced ICH-induced neuronal apoptosis and oxidative stress. LKLF: Lung-Kruppel-like factor; Mfn2: mitofusin2; ROS: reactive oxygen species; MDA: malondialdehyde; GSH: glutathione.

In summary, we discovered that acupuncture treatment might stimulate LKLF expression, which activated Mfn2 transcriptional activity, alleviated mitochondrial injury, and reduced ICH-induced neuronal apoptosis and oxidative stress (Figure 7). This provides a strong foundation for elucidating the molecular mechanism of acupuncture in the treatment of ICH, presents a novel prospective target for ICH treatment, and is important for identifying the function of the LKLF/Mfn2 pathway.

## Data Availability

All data generated or analyzed during this study are included in this published article.
